# Vascular surgery of aortic thrombosis in a dog using Fogarty maneuver – technical feasibility

**DOI:** 10.1002/ccr3.1295

**Published:** 2017-12-16

**Authors:** Maartje Schwede, Olaf Richter, Michaele Alef, Tobias Theuß, Shenja Loderstedt

**Affiliations:** ^1^ Department of Small Animal Medicine University of Leipzig Leipzig Germany; ^2^ Ev. Diakonissen‐Krankenhaus Leipzig Germany; ^3^ IDT Biologika GmbH BU Animal Health, Research and Development Dessau‐Rosslau Germany

**Keywords:** Aortic thromboembolism, Canid, cardiovascular disorders, vascular surgery

## Abstract

Aortic thromboembolism is a rare and life‐threatening disease in dogs. This report aims to describe the successful surgical treatment by use of a Fogarty Thrombectomy Catheter in an 8‐year‐old patient. The postsurgical intensive care therapy to prevent ischemia‐reperfusion syndrome is specified, despite poor outcome in our case (owner elected euthanasia).

## Introduction

Aortic thrombosis is a rare finding in dogs 1, 2, 3, 4, 5, 6, 7 and usually occurs at a mean age of 8.3 years 3. In contrast to cats where myocardial hypertrophy is the predominant etiology, neoplastic diseases, hyperadrenocorticism, hypothyroidism, protein‐losing enteropathy, hemolytic anemia, disseminated intravascular coagulation, and glomerulonephritis are reported as causative for aortic thrombosis in dogs 2, 3, 4, 7, 8, 9, 10. Different options are described for treatment of aortic thrombosis in veterinary medicine 6, 7, 11. We describe a clinical severe case surgical successfully treated with Fogarty maneuver.

## Case Presentation

### Signs and symptoms

An eight 8‐year‐old male Australian Shepherd (27 kg) was presented to our veterinary teaching hospital with a history of apathy and weakness of the pelvic limbs occurring within the last 4 days. Initial therapy with amoxicillin and methylprednisolone in a not further defined dosage was given by the referring veterinarian without improvement of the clinical symptoms. Initial examination at our veterinary teaching hospital revealed a mild disturbed general condition, paraplegia, loss of voluntary bladder control, and the absence of femoral pulse bilaterally. Temperature of both pelvic paws was assessed manually subjective and evaluated not different between the left and the right.

Neurological examination revealed normal mentation and generalized weakness and paraplegia without deep pain perception in both pelvic limbs. The cranial nerve examination showed slight reduced sensibility and was otherwise within normal limits. The proprioception and flexor reflexes were slightly reduced in the thoracic and absent in the pelvic limbs. The patellar tendon reflex was reduced bilateral. Deep pain perception was normal in the thoracic and absent in the pelvic limbs. Neuroanatomical localization to one lesion was not possible. Due to the severity of symptoms of the pelvic limbs, a L4‐S3 spinal cord segments or neuromuscular lesion of the pelvic limbs in conjunction with a systemic disease or C6‐T2 myelopathy was considered. The absent femoral pulse was highly suggestive for an ischemia of both pelvic limbs.

### Diagnostics

A sonographic examination of the abdomen revealed an inhomogeneous, medium intense structure in the *Aorta abdominalis* between *A. mesenterica caudalis* and the bifurcation into the *Aa. iliacae externae*, consistent with aortic thrombosis. Computed tomography angiography was performed under generalized anesthesia and confirmed this diagnosis. Coagulation indices prothrombin time and thromboplastin time were within normal range. Hematology prior anesthesia revealed a mild leucocytosis 23.63 × 10^9/L (5.05–16.76), increased ALP 502 U/L (13–83), ALT 873 U/L (17–78), and BUN 10.63 mmol/L (3.28–10.42). The urine was tar‐like discolored, with positive severe increase in protein, bilirubin, and hemoglobin (Combur^9^‐Test^®^, Roche, Rotkreuz, Switzerland); urine protein/creatine ratio was elevated 1.32 (0–0.5). Angiostrongylus vasorum antigen snap test was negative.

### Therapy and perioperative management

After informed consent of the owners, a surgical thrombectomy was performed. For general anesthesia, the dog received intravenous (i.v.) premedication with 0.25 mg/kg levomethadone (L‐Polamivet^®^, MSD, Unterschleißheim, Germany), 3.0 mg/kg ketamine (Ketamin 10%, Bremer Pharma GmbH, Warburg, Germany), and 0.5 mg/kg diazepame (Diazepam 10 MG, Rotexmedica, Trittau, Germany). Anesthesia was induced with 4 mg/kg bolus of intravenous propofol (Narcofol^®^ 10 mg/kg, cp‐pharma, Burgdorf, Germany) and maintained with isoflurane (1–2%) in an oxygen‐air mixture (1:2) and a lidocaine–ketamine CRI (lidocaine 1 mg/kg/h after loading dose of 2 mg/kg i.v. [Xylocitin‐cor 1%, Mibe GmbH, Brehna, Germany], ketamine 1 mg/kg/h,), both diluted in Ringer‐acetate solution (B. Braun Melsungen AG, Melsungen, Germany, infusion rate 5 mL/kg/h). The dog was ventilated with a volume‐controlled IPPV (tidal volume 400 mL, respiratory frequency 9–12/min, target endtidal CO_2_‐concentration 4.5–5.5 Vol.‐%, resulting PIP 14 cmH_2_O). Continuous monitoring included clinical evaluation, in‐ and expiratory concentrations of CO_2_, O_2_, and isoflurane, patient spirometry, ECG, pulse oximetry, esophageal temperature, and NIBP on the right thoracic limb. Voluven^®^ 6% (Fresenius Kabi, Bad Homburg, Germany) was used to compensate blood loss.

### Surgical procedure

**Figure 1:**
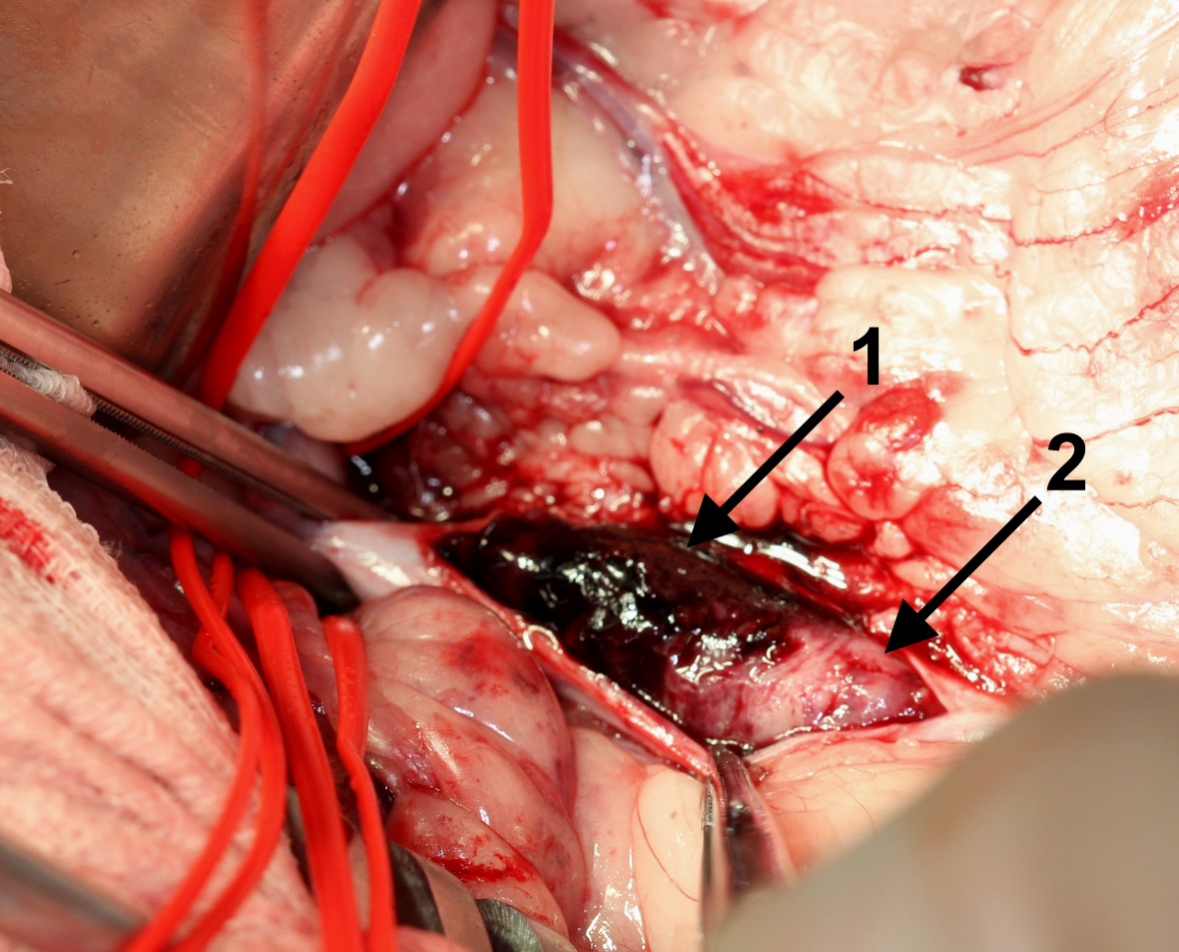
Surgical preparation of the thrombus after incision of the aortic wall. Note parts of red thrombus with predominance of erythrocytes (1) and white thrombus formation with predominance of fibrin and platelets (2).

**Figure 2:**
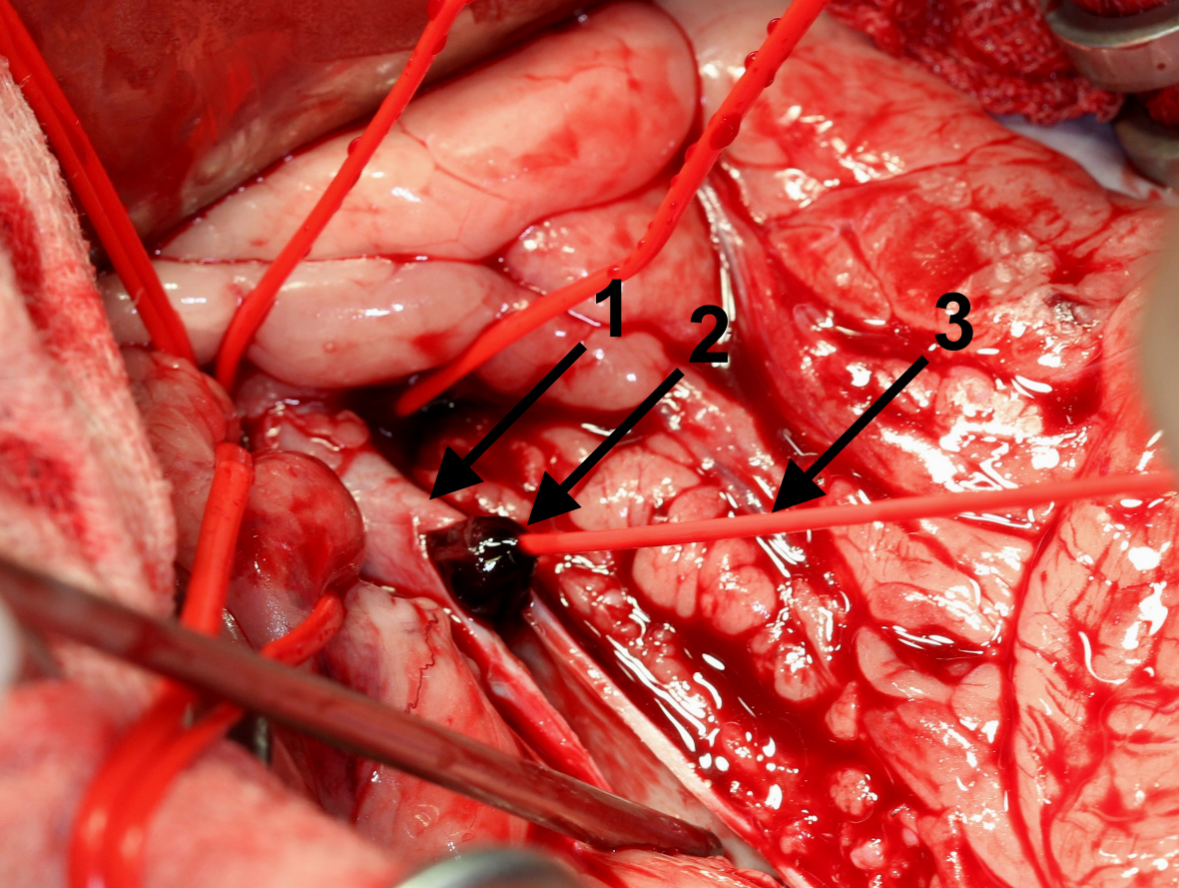
Demonstration of the thrombectomy via pulling the thrombus (1) out of the aorta (2) using the Fogarty catheter (3).

**Figure 3:**
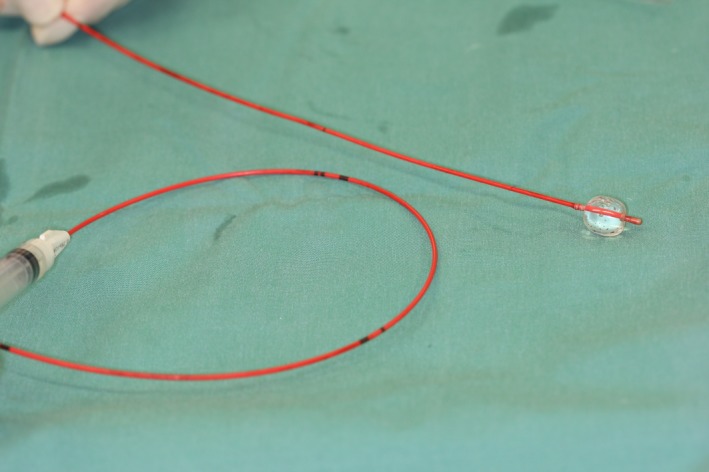
Fogarty catheter with filled balloon to retract thrombus.

The aorta was exposed by opening of the abdomen in the *Linea alba,* replacement of the abdominal organs with a retractor, and opening of the retroperitoneal space. A ‘Rummel Tourniquet’ (Argyle™ Vascular Tourniquet Kit, Covidien, Dublin, Ireland) placed approx. 2 cm cranial of the thrombus (caudal of the *A. mesenterica caudalis*) was used to suppress aortic blood flow cranial to the thrombus. Two minutes prior to aortotomy, the dog received 110 IE/kg heparin intravenously (Heparin‐Natrium 5000 IE, Ratiopharm, Ulm, Germany). The aorta was incised longitudinal approximately 3 cm (Fig. 1). Major parts of the thrombus (Fig. 1) were removed with the forceps. For extraction of the remaining thrombus, a 4 F Fogarty^®^ ‘Arterial Thrombectomy Catheter’ (LeMaitre^®^ Single Lumen Embolectomy Catheter, Burlington, VT, balloon diameter 10.5 mm, balloon volume 0.75 mL, length 80 cm) was used (Fig. 2). For this, the catheter was inserted in both directions through the thrombus to the end of the vessel (about 20 cm). The catheter balloon was insufflated and the catheter pulled back. This manoeuver enables to retract all thrombotic material from distal and proximal areas of the affected vascular region of the *Aa. iliacae externae* and *Aa. Iliacae internae* (Fig. 3). Thrombus adhesion to the aortic wall was not apparent. Remaining small particles were flushed out of the vessel with physiological sodium chloride solution. The aorta was closed with a continuous suture (Polypropylene, Prolene^®^ 4/0, Somerville, MA) and the abdomen according to standard procedures. Noninvasive blood pressure was measured on the right forelimb during surgery and was within normal limits throughout. A normal pulse was palpable over the *A. femoralis* bilateral after the procedure. During surgery, the dog lost about 500 mL blood resulting in a decline in hematocrit from 55.2% prior surgery to 30.0% immediately postsurgery.

### Histopathology

**Figure 4:**
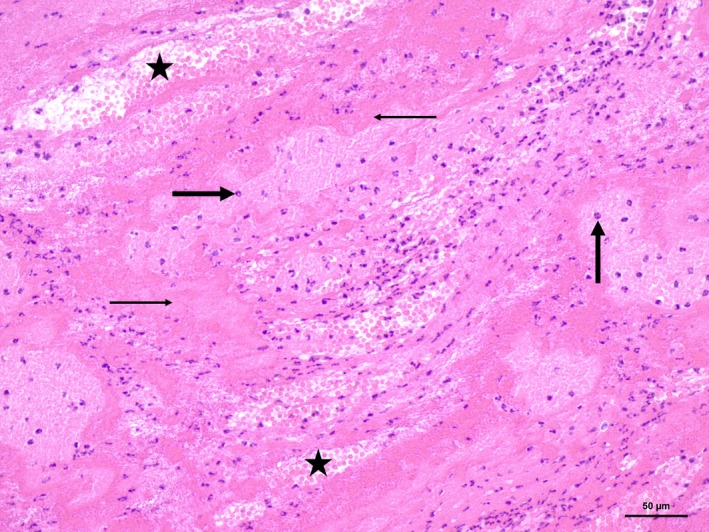
The thrombus was composed of a fibrin scaffold with interlaced areas of erythrocytes (asterisks), proteinaceous eosinophilic material (thin arrows), and multifocal varying amounts of neutrophilic granulocytes (thick arrows). No histological sings of thrombus organization, for example, fibroblast proliferation (granulation tissue) or re‐endothelization, were obvious. Hemalum‐eosin staining, Bar 50 *μ*m.

Six representative localizations of the thrombus were examined histopathologically (H.‐E.‐staining of formalin‐fixed paraffin‐embedded specimens). The thrombus was composed of a fibrin mesh with interlaced numerous erythrocytes, occasional neutrophils, lymphocytes, and monocytes. A formation of granulation tissue indicative for organization of the thrombus was not found, and no bacteria or neoplastic cells were obvious (Fig. 4).

### Clinical course and outcome

The dog was monitored half‐hourly during the first 24 h after surgery. He presented in lateral recumbency with severely edematous pelvic limbs, pale mucous membranes, and tachypnoe (>60/min). A strong, regular femoral pulse was palpable bilateral. Over the first 24 h postsurgery moderate leucocytosis (27.8 × 10^9/L), nonregenerative anemia (hematocrit 21.1%) and thrombocytopenia (124 K/*μ*L), hypoproteinemia (34 g/L), hypalbuminemia (10 g/L), hyperkalemia (6.7 mmol/L), and azotemia (urea 16.98 mmol/L, creatinine 199 *μ*mol/L) occurred. The dog received Ringer‐acetate CRI 4–6 mL/kg/h intravenous (Baxter, Deerfield, IL), propentofyllin oral 100 mg (Karsivan^®^, MSD, Unterschleißheim, Germany) and for pain control levomethadone 0.25 mg/kg subcutaneous, metamizol sodium 50 mg/kg intravenous (Metapyrin^®^, Serumwerk Bernburg AG, Bernburg, Germany), gabapentin 4 mg/kg oral (Gabapentin HEXAL^®^, Hexal AG, Holzkirchen, Germany). For treatment of peripheral edema, furosemide 2 mg/kg intravenous (Dimazon^®,^ MSD, Unterschleißheim, Germany) and dexamethasone 0.11 mg/kg intravenous (Dexamethason, bela‐pharm, Vechta, Germany) were given. To prevent intravascular coagulation, enoxaparin sodium CRI 3.34 mL/h intravenous (Clexane^®^ multidose, Sanofi‐Aventis, Frankfurt am Main, Germany) was given. Unfortunately, the edema and dyspnoea increased further during the course of the day and owner decided for euthanasia.

## Discussion

The dog presented here showed clinical symptoms highly indicative for aortic thrombosis 1, 3, 4, 5, 6, 9, 10, 12, 13 which started approximately 4 days prior presentation. For acute ischemia in humans, Pratt et al. stated: “It can be diagnosed usually on the history alone. The signs paleness, pulselessness, paraesthesia, paralysis, pain and prostration are apparent.” 14. Except the paleness, which has not been particularly documented, all other of these so‐called six‐P‐signs were present in this case. The clinical presumptive diagnosis was confirmed via ultrasound and computed tomography, both suitable methods for definite diagnosis 7, 11.

Three major predispositions of thrombosis have to be contemplated 15: (1) hypercoagulability, (2) altered blood flow (e.g., through dilative cardiomyopathy), and (3) injury of the endothelium. As the exact cause of thrombosis cannot be identified in most cases, they are usually referred as “idiopathic.” Considering the histopathology of the thrombus, a vessel eroding neoplasia, chronic vascular endothelial disease, or hematogenous bacterial infection (e.g., endocarditis) as a possible etiology for thrombus formation seems unlikely in this case. Due to the absence of granulation tissue, a short‐term history of disease is likely consistent with the clinical history and presumptive diagnosis of aortic thrombosis. Possible emboli origins in humans are the left atrium, endocarditis, dilative cardiomyopathy, acute myocardial infarct, different forms of aneurisms, paradox emboli, compression, or paraneoplastic syndrome 16. The owner never noticed any signs of cardiac disease, and no abnormal heart murmur was noticed during clinical and pre‐anesthetic examination of the patient. However, a complete rule out of other possible causes of thrombi formation would have required a full necropsy of the dog, which was denied by the owner.

In humans, acute ischemia of extremities with an incidence of 7–15/100,000 per year appears in 85% of the cases in lower extremities (equivalent to pelvic limbs) 17. The pathogenesis and presentation of aortic thrombosis in dogs are not well characterized 6. A recent review concerning aortic thrombosis in dogs discriminates aortic thromboembolism and aortic thrombosis 7. For canine patients with aortic thrombosis kidney disease (9/18) and artherosclerosis associated with hypothyroidism (4/18) are described as common concurrent conditions in a retrospective pathological study 4. The dog presented here did not have history of kidney disease and hypothyroidism, and no artherosclerotic changes were noticed during surgery. Another study of 26 dogs with aortic thrombosis describes an improvement of ambulatory function in all of the 14 dogs treated with warfarin protocol. However, 20 of 26 dogs were ambulatory 6. There is evidence in veterinary literature that acutely nonambulatory dogs with aortic thrombosis have a poor prognosis 18. For human patients, ischemia of lower extremities can be graded I to III by Rutherford 19. Grade Rutherford III is classified with loss of pain perception, paralysis and loss of arterial, and venous Doppler signal and caused mayor tissue loss or permanent nerve damage and is an indication for surgical intervention. Limb amputation is often the consequence. In our case, the clinical severity was equivalent to a grade III lesion. Considering the severity and both pelvic limbs being affected, conservative treatment or limb amputation was not an alternative.

Different surgical interventions for thrombectomy or an endovascular thrombolysis are described in the human medical literature 20, 21. In dogs, peripheral vascular disease is uncommon 6, and therefore, a Fogarty maneuver was adequate for thrombectomy. In our case, there was no evidence of any macroscopic disease process involving the arterial wall. With the Fogarty technique, a wired catheter is pushed passed the arterial thrombus and by pulling back after inflating the balloon on the catheter tip, the thrombus can be retrieved. Advantage of this method is a small arteriotomy and a relatively long distance of possible endovascular thrombectomy.

One of the major postoperative risks in acute extremities ischemia cases after successful revascularization is the reperfusion syndrome, causing metabolic acidosis, rhabdomyolysis, myoglobin urea, and acute renal failure 22. The reperfusion syndrome causes swelling of muscles leading to compartment syndrome. In human medicine, intramuscular pressure monitoring, and fasciotomy are advised in these patients 16. In the presented case, severely edematous pelvic limb swelling and acidosis occurred the day after surgery indicating reperfusion syndrome. During the postischemic phase‐reactive oxygen species, toxic metabolites, lactate, and hydrogen protons build up causing endothelial damage and leakage of albumin into the tissue which increases tissue edema and in contrast decreases plasma albumin 22, 23. The latter was also seen in our case. Unfortunately, in this case, LDH, CK, and myoglobin level were not evaluated, due to financial restrictions by the owner. However, the tar discolored urine likely was consistent with myoglobinuria and rhabdomyolysis.

In humans, the 30‐day mortality rate after a vascular open surgical intervention is 4.4–22% 16. The postsurgical complication rate was 15.1% in one study 24. In a different study, fasciotomies had to be performed in 39% and unexpected return to operating room occurred in 24% of cases 25.

In the dog described in this report, further diagnostic and therapeutic procedures were declined by the owner on the day after surgery, and the dog unfortunately was euthanized upon request of the owner. The postsurgical therapy applied in our case was overall aligned to the recommendations of treatment in humans 20, including appropriate medical therapy (in particular heparin) and closely clinical monitoring. For evaluation of the re‐established blood flow, the skin surface could be monitored quantitatively by use of an, for example, surface thermometer, transcutaneous oxygen pressure measurement, or skin perfusion pressure measurement 26, and not only manually as we did. However, due to the dense hair coat in canine patients, surface thermometers are less reliable in animals compared to humans 27, 28. A final assessment of a potential treatment outcome is not possible in our case, as the decision for euthanasia took place about 24 h after the surgery. Without the owner's choice, the outcome in this particular case could have been probably better. However, we have to accept this in veterinary medicine, as final treatment decision is made by the owner influenced by personal ethical understanding of animal welfare. Furthermore, the costs for treatment have to be paid by the owners themselves, if as in our case, there is no health insurance for the animal.

Acute aortic thrombosis should be considered as differential diagnosis in dogs presenting with paraplegia and can easily be verified by the absence of femoral pulse. A thrombectomy by Fogarty maneuver as surgical method is technical moderate challenging and therefore can very well be used as treatment option in dogs suffering from this disease. Although this technique is quite uncommon in veterinary medicine, further training of veterinary surgeons by human vascular surgeons is necessary. Postsurgical management is likely to be crucial for the outcome of such cases. Therefore, the owner of such patients needs to intellectually appreciate the complexity of the disease and the need for intensive and critical care postsurgery, as well as the costs joint with it. More information is needed to give objective prognoses for surgical therapy of clinical severe aortic thrombosis in dogs.

## Authorship

MS: assistant surgeon (veterinarian) during surgery, wrote the manuscript. OR: main surgeon who performed surgery (medical doctor, specialist in vascular diseases), revised the manuscript. MA: veterinary anesthesiologist, revised the manuscript. TT: certified veterinary pathologist, co‐authored the manuscript. SL: assistant surgeon (veterinarian), co‐authored the manuscript.

## Conflict of Interest

None declared.
